# Salt-dependent Blood Pressure in Human Aldosterone Synthase-Transgenic Mice

**DOI:** 10.1038/s41598-017-00461-9

**Published:** 2017-03-28

**Authors:** Huiying Gu, Zhizhong Ma, Jian Wang, Timothy Zhu, Nicole Du, Adam Shatara, Xin Yi, Mark C. Kowala, Yansheng Du

**Affiliations:** 1 Department of Neurology, Indiana University School of Medicine, Indianapolis, IN USA; 2 Basic Medical School, Peking University, Beijing, China; 3 Lilly Research Laboratories, Eli Lilly and Company, Indianapolis, IN USA

## Abstract

Hypertension is one of the most important, preventable causes of premature morbidity and mortality in the developed world. Aldosterone is a major mineralocorticoid hormone that plays a key role in the regulation of blood pressure and is implicated in the pathogenesis of hypertension and heart failure. Aldosterone synthase (AS, cytochrome P450 11B2, cyp11B2) is the sole enzyme responsible for the production of aldosterone in humans. To determine the effects of increased expression of human aldosterone synthase (hAS) on blood pressure (BP), we established transgenic mice carrying the hAS gene (cyp11B2). We showed that hAS overexpression increased levels of aldosterone in hAS^+/−^ mice. On high salt diet (HS), BPs of hAS^+/−^ mice were significantly increased compared with WT mice. Fadrozole (an inhibitor of aldosterone synthase) treatment significantly reduced BPs of hAS^+/−^ mice on HS. This is the first time overexpression of AS in a transgenic mouse line has shown an ability to induce HP. Specifically inhibiting AS activity in these mice is a promising therapy for reducing hypertension. This hAS transgenic mouse model is therefore an ideal animal model for hypertension therapy studies.

## Introduction

Hypertension (HP) is a polygenic and multifactorial disease that has high incidence not only in the developed countries, but also in developing countries. Researchers have found that the number of adults with hypertension more than doubled from 1995 to 2005^1^. Furthermore, Kearney and colleagues predict that there will be a relative increase of 24% in the prevalence of hypertension in developed countries from 2000 to 2025^2^.

Aldosterone, the principal mineralocorticoid in humans, is produced in the zona glomerulosa of the adrenal cortex by aldosterone synthase (CYP11B2). As a key component of the renin-angiotensin-aldosterone system, aldosterone acts primarily at the renal distal convoluted tubules as a critical regulator of fluid and electrolyte homeostasis. The primary secretagogues that stimulate aldosterone biosynthesis are angiotensin II (AngII), adrenocorticotropic hormone (ACTH), and potassium. They all elevate cytoplasmatic calcium levels and promote activation of steroidogenic enzymes including aldosterone synthase (AS or 18-hydroxylase)^3^. It suggests that AS encoded by the CYP11B2 gene may contribute to dysregulation of aldosterone synthesis^4, 5^.

Conn initially described that primary aldosteronism (PA) proved the existence of a direct relationship between elevated levels of aldosterone and the development of hypertension^6^. Recent data indicated that PA is present in up to 15% individuals with hypertension^7, 8^. Given the roles that aldosterone plays in causing hypertension and promoting vascular, renal, and myocardial disease^9–12^, blocking aldosterone production could be one option for lowering blood pressure (BP) and mitigating the target-organ damage associated with hypertension^13, 14^. Inhibition of aldosterone synthase is currently being investigated as a medical treatment for hypertension, heart failure, and renal disorders^15^. For PA, with the exception of the small proportion of patients with familial hyperaldosteronism type I, the underlying genetic and molecular basis of the disease remains largely unknown, particularly the role of aldosterone synthase in the pathogenic mechanism of primary aldosteronism-related hypertension is currently not quite clear. Two reported AS transgenic mouse lines have demonstrated either no or modest 10-mm Hg higher blood pressure on the high-salt diet than on the low-salt diet (117 ± 3 mm Hg vs. 107 ± 3 mm Hg) and wild-type mice on either diet^16, 17^. Therefore, it is necessary to develop a new AS transgenic mouse line that can demonstrate aldosterone-mediated HP symptom by markedly inducing BP in mice treated with high salt. Development of experimental models of PA and hypertension allow dissection and isolation of various factors associated with regulation of blood pressure, inheritance of hypertensive traits, and cellular responses to injury. The mouse model with AS overexpression can be used to confirm and define functional relevance of the hAS gene that are known or suspected to be involved in the regulation of aldosterone secretion and HP. Moreover, the AS transgenic mouse model can aid in the identification of novel gene products that have not yet been identified to play a role in PA and hypertension.

In this study, we have successfully established a transgenic mouse model carrying human aldosterone synthase gene (cyp11B2) under the control of cyp11B1 promoter and found increased expression of hAS induces BP in this mouse. Significantly high serum levels of human aldosterone and high-salt-induced hypertension were observed in this mouse model. Interestingly, the serum level of aldosterone and high-salt-induced hypertension could be reduced by fadrozole in these mice. Our data suggest that AS levels are likely to affect BP responds to changes in dietary salt and aldosterone, and this hAS overexpression mouse line may be a useful model to study AS-mediated PA, hypertension, and possibly chronic kidney disease and heart failure.

## Results

### Generation of *cyp11B1-hAS* transgenic mice

**Figure 1:**
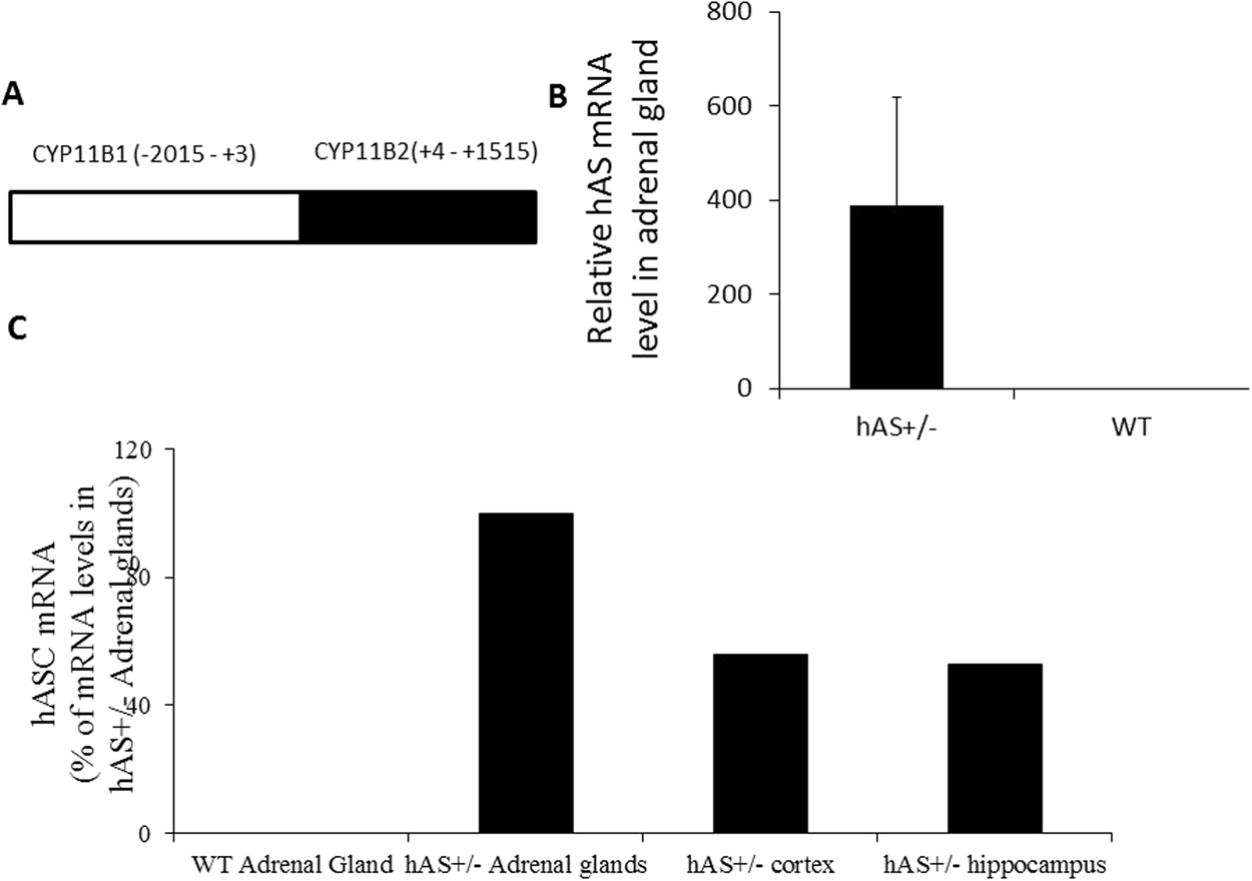
Generation of hAS transgenic mice. (**A**) Scheme of the cyp11B1-hAS fragment containing the human cyp11B1 promoter (pCYP11B1 (−2015–+3)) and the human hAS coding sequence (cDNA, +4–+1515). (**B**) Detection of hAS mRNA levels in adrenal glands of hAS^+/−^ transgenic mice by RT-PCR. (**C**) Detection of hAS mRNA levels in adrenal gland, brain cortex and hippocampus of hAS^+/−^ transgenic mice by RT-PCR.

To study whether AS was a therapeutic target for PA-related hypertension, we generated a transgenic mouse line expressing the hAS coding region (+4–+1115) under the control of the human cyp11B1 promoter (pCYP11B1 (−2015–+3))^18^ (Fig. 1A). This promoter was shown to target gene expression in the adrenal cortex^19, 20^. Figure 1B showed a ~400-fold increase of the hAS mRNA in adrenal glands of hAS^+/−^ mouse compared with age-matched WT mouse. The significant amounts of hAS mRNA were also detected in the brain cortex (56% of mRNA levels present in adrenal glands) and hippocampus (53% of mRNA levels in adrenal glands) of hAS^+/−^ mouse (Fig. 1C).

### General Characteristics of hAS^+/−^ and WT Mice

All hAS^+/−^ mice showed normal health, growth, and fertility for a year (average 6 pups/litter, ~40 breeding pairs in Indiana University) as compared to WT mice. Postnatal development and basic phenotype parameters were similar in both TG line and wild type mice (n > 100/group). The results also showed that differences in the genotypes did not significantly affect weights of body, adrenal gland, heart, and kidney (n = 8/group, Table 1).

**Table 1:** General Characteristics of hAS^+/−^ and WT Mice.

	WT (n = 8)	TG (n = 8)
Body weight (g)	45.8 ± 3.7	42.7 ± 3.6
Heart weight (mg)	143.1 ± 19.6	148.1 ± 21.9
Heart wt/BW (mg/g)	3.5 ± 0.4	3.1 ± 0.5
Kidney weight (mg)	54.8 ± 12.7	56.1 ± 11.6
Kidney wt/BW (mg/g)	12.0 ± 3.0	13.2 ± 2.6
Adrenal Gland weight (mg)	5.0 ± 1.0	4.7 ± 1.0
Adrenal gland wt/BW (mg/g)	0.11 ± 0.03	0.11 ± 0.02

The high expression of hAS gene in hAS^+/−^ mice did not affect body weight, adrenal gland weight, heart weight, and kidney weight.

### hAS overexpression increased levels of aldosterone in hAS^+/−^ mice

**Figure 2:**
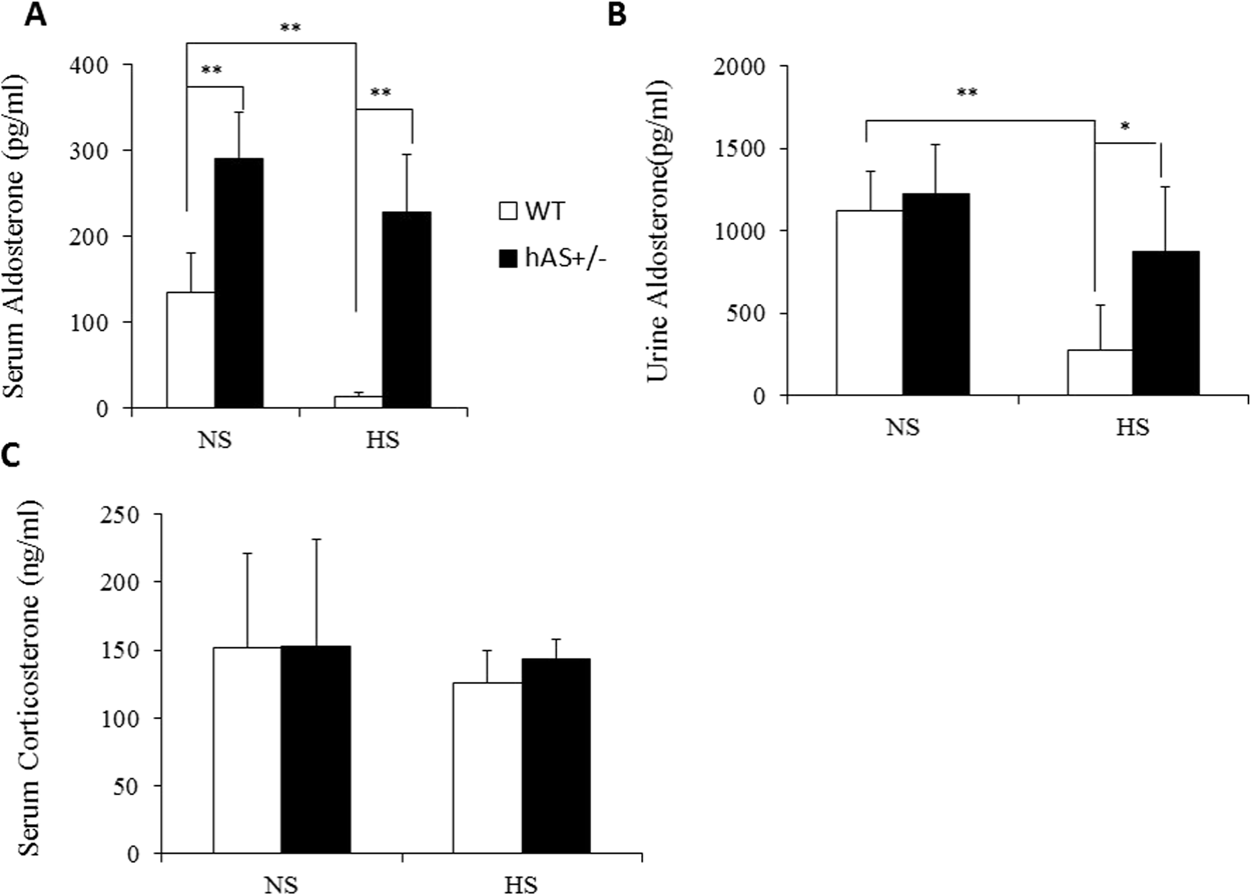
hAS overexpression increased levels of aldosterone in hAS^+/−^ mice. (**A**) The serum aldosterone levels of WT and hAS^+/−^ mice on NS or HS were detected by ELISA. (**B**) The levels of urine aldosterone were tested by ELISA as well. (**C**) The serum corticosterone levels of WT and hAS^+/−^ mice on NS or HS were measured by ELISA. *p < 0.05; **p < 0.01. NS: normal salt diet; HS: high salt diet.

In order to check the effect of hAS overexpression on the levels of aldosterone in mice, the serum and urine levels of aldosterone were tested by ELISA. On normal sodium diet (NS), the serum level of aldosterone in hAS^+/−^ mice was 299.0 ± 67.2 pg/ml, increased markedly (~2-fold) as compared to 123.8 ± 38.7 pg/ml in WT mice (n = 4, p < 0.01). High sodium diet (HS, 4% NaCl, Harland Teklad (2018)) suppressed serum level of aldosterone significantly in WT mice to 12.4 ± 6.3 pg/ml (n = 4, p < 0.01). However, serum aldosterone level was slightly decreased in HS hAS^+/−^ mice (227.7 ± 67) but without statistical significance and it was much greater than HS WT mice (Fig. 2A). On NS, the urine levels of aldosterone in WT (1123.3 ± 234.9 pg/ml) and hAS^+/−^ (1227.7 ± 297.1 pg/ml) mice were similar. Urine aldosterone level of WT mice with HS was significantly reduced to 277.3 ± 276.4 pg/ml. However, hAS overexpression suppressed HS-induced urine aldosterone reduction in hAS^+/−^ mice (877.2 ± 389.1 pg/ml) (Fig. 2B).

Corticosterone and aldosterone share the first part of their biosynthetic pathways. The last part are mediated either by the aldosterone synthase (for aldosterone) or by the 11β-hydroxylase (for corticosterone). To test the effect of hAS overexpression on the level of corticosterone, the serum level of corticosterone was tested by ELISA. The serum corticosterone levels did not show significant changes under hAS overexpression with either normal sodium diet or high sodium diet (Fig. 2C).

### High-salt induced hypertension in hAS^+/−^ mice

**Figure 3:**
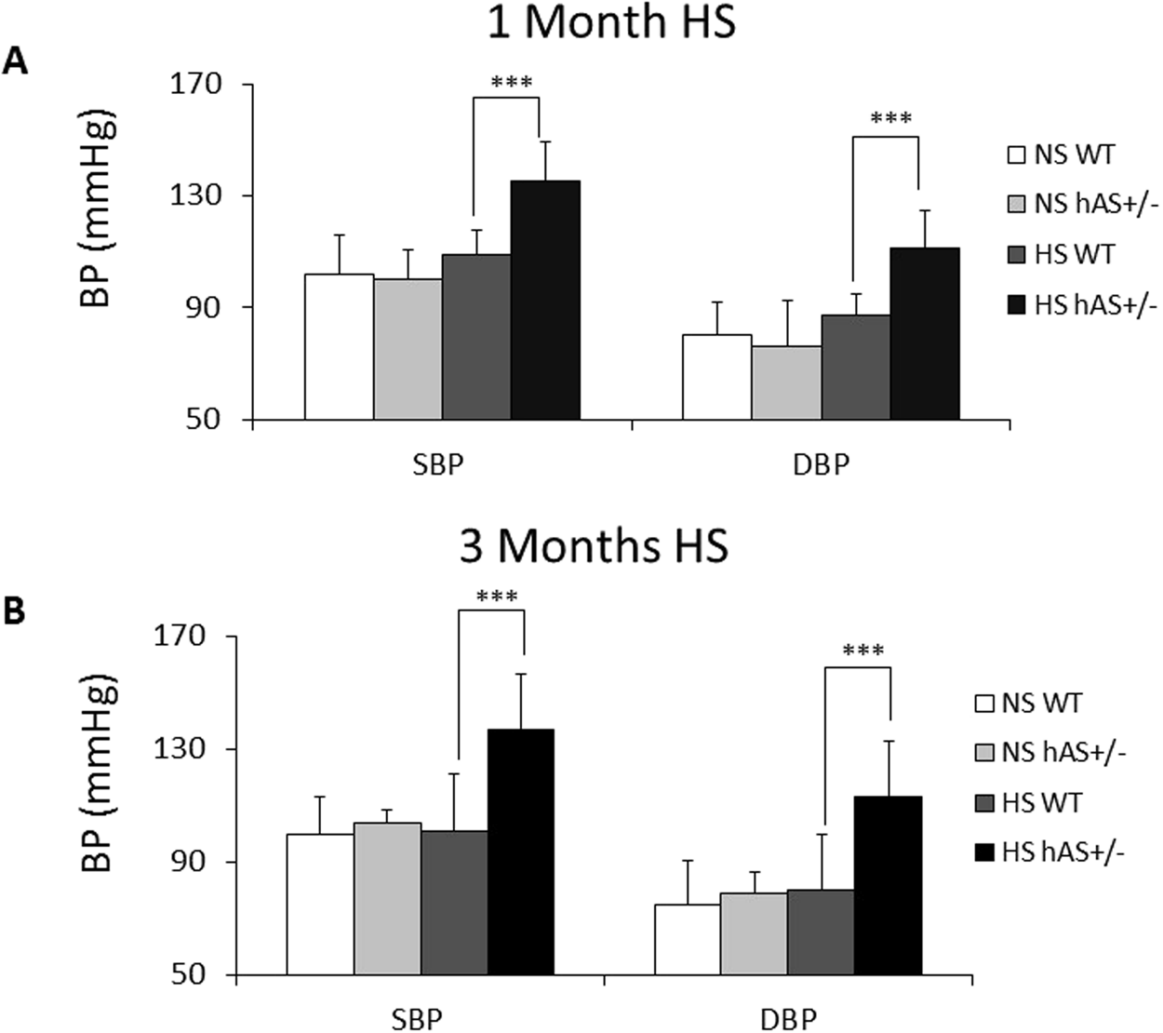
High-salt induced hypertension in hAS^+/−^ mice. (**A**) On 1 month treatments with HS or NS diets, BPs of WT and hAS^+/−^ mice were measured. (**B**) Continued on HS or NS for a total of 3 months, BPs of WT and hAS^+/−^ mice were detected. ***p < 0.001. SBP: systolic blood pressure; DBP: diastolic blood pressure.

The altered salt diets (NS, and HS) did not significantly affect the BPs of WT mice (Fig. 3). In contrast, the dietary salt affected the BPs of hAS^+/−^ mice. On a 1 month treatment with the HS diet, the BPs of hAS^+/−^ mice were significantly increased compared with WT mice (SBP: 135 ± 14.1 mmHg versus 109 ± 8.8 mmHg; DBP: 111 ± 13.9 mmHg versus 87 ± 8.0 mmHg, P < 0.001, n = 9) (Fig. 3A). With the continuation of the HS diet for a total of 3 months, BPs of hAS^+/−^ mice were still significantly higher than WT mice (SBP: 137 ± 12.6 mmHg versus 101 ± 9.1 mmHg; DBP: 113 ± 19.5 mmHg versus 80 ± 8.3 mmHg, P < 0.001, n = 9) (Fig. 3B).

### Electrolytes, renin, hematocrit, urine pH, and kidney gene expressions in WT and hAS^+/−^ mice

In order to further characterize the hAS^+/−^ mice, we examined plasma and urinary levels of Na^+^ and K^+^ in both WT and AS^+/−^ mice (Table 2). As expected, plasma levels of Na^+^ were significantly higher in hAS^+/−^ mice as compared to WT mice. In contrast, plasma levels of K^+^ were significantly lower in hAS^+/−^ mice as compared to WT mice. Although HS diets did not increase plasma levels of Na^+^ in both hAS^+/−^ and WT mice, in hAS^+/−^ mice, HS significantly reduced K^+^ levels in hAS^+/−^ mice. Additionally, consistent to previous report^21^, plasma renin activities in both hAS^+/−^ and WT mice were markedly decreased in HS fed mice as compared to NS fed mice. In both NS and HS fed mice, hAS^+/−^ showed significantly higher plasma renin activity than WT mice. There were slightly but significant reduced hematocrit in NS fed hAS^+/−^ mice as compared to NS fed mice and HP induced by HS was accompanied with significantly increased hematocrit in hAS^+/−^ mice as compared to WT mice without induced HP. In urine, hAS^+/−^ mice showed significantly reduced levels of Na^+^ than WT mice. As predicted, HS stimulated Na^+^ excretions and decreased urine potassium excretion in both types of mice. Since more Na^+^ was secreted in HS-fed WT mice, urines from these mice showed significantly higher pH than NS-fed WT and both NS- and HS-fed hAS^+/−^ mice.

**Table 2:** Plasma electrolytes and renin activity as well as urinary excreted electrolytes and pH in AS^+/−^ and WT mice ﻿treated with NS and HS diets﻿.

Electrolytes		WT	AS^+/−^
Plasma			
Na^+^, mmol/L	NS	147 ± 0.51 (5)	151 ± 0.32 (15)^†^
	HS	148 ± 0.51 (5)	152 ± 0.69 (15)^§^
K^+^, mmol/L	NS	4.8 ± 0.06 (5)	4.5 ± 0.07 (15)^†^
	HS	4.88 ± 0.08 (5)	4.0 ± 0.06 (15)*^,§^
Renin activity	NS	5.19 ± 0.25 (4)	1.7 ± 0.31 (15)^†^
	HS	285 ± 43.73 (5)	208 ± 16.5 (15)^§^
Urine			
Na^+^, mmol/L	NS	125 ± 16.99 (5)	95 ± 8.17 (15)^†^
	HS	285 ± 43.73 (5)	208 ± 16.5 (15)^§^
K^+^, mmol/L	NS	180 ± 20.46 (5)	134 ± 15.97 (15)^†^
	HS	62 ± 11.68 (5)	71 ± 7.16 (15)*^,§^
PH	NS	7.0 (5)	6.9 (14)
	HS	7.7 (5)^#^	7.0 (15)

Values are means ± SE and numbers in parentheses represent numbers of used animals. *p < 0.05 HS versus NS in AS^+/−^ groups, ^†^ *p<*0.05, AS^+/−^ versus WT with NS; ^§^ *p*<0.05, AS^+/−^ versus WT with HS; ^#^ *p* < 0.05, HS versus NS in WTgroups.

**Figure 4:**
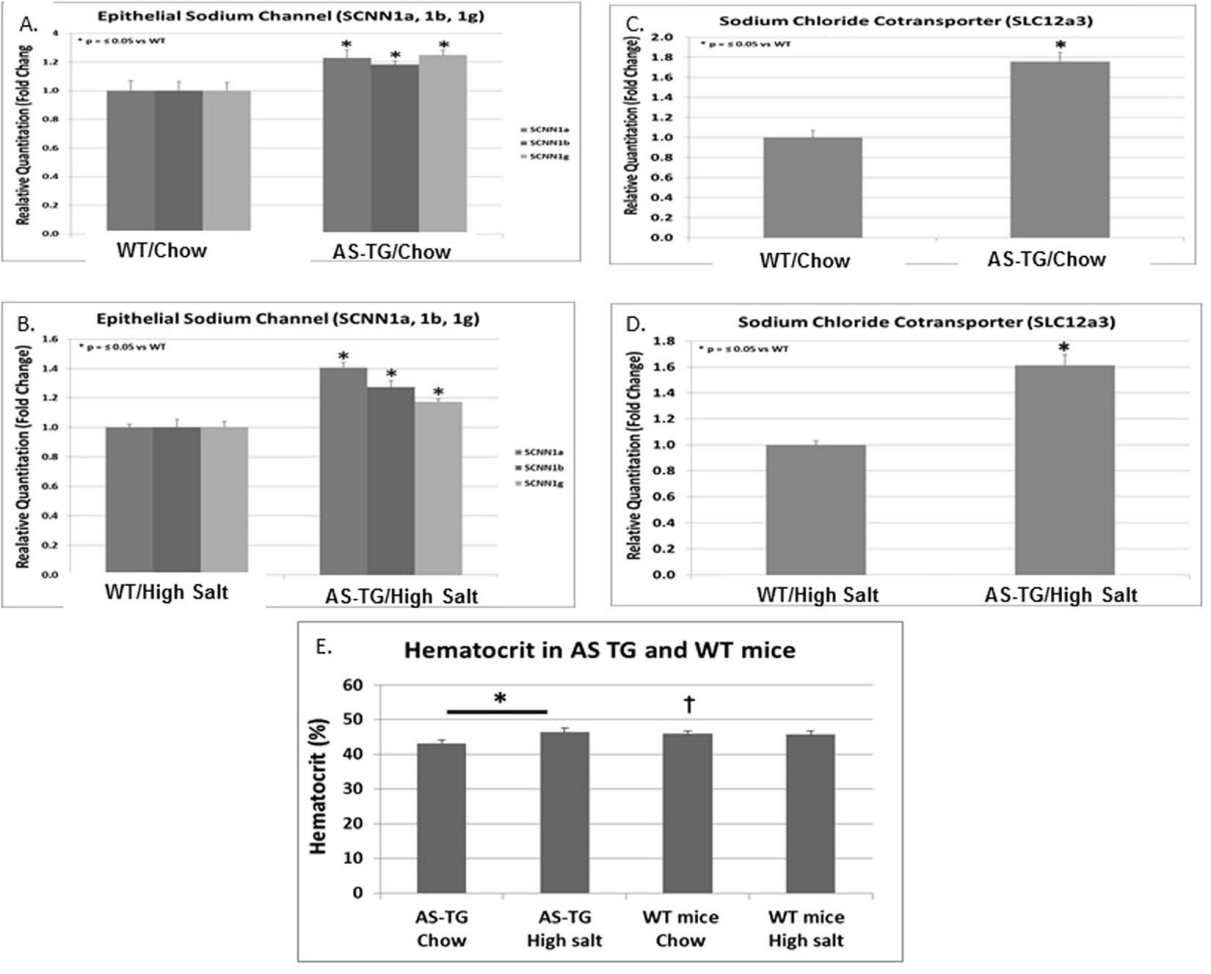
Kidney gene expressions and hematocrit in WT and hAS^+/−^ mice. (**A**,**B**) hAS^+/−^ (AS-TG) mice showed significantly higher gene expressions of the three kidney ENaC subunits encoded by SCNN1a, SCNN1b, and SCNN1g compared to WT mice fed with either NS or HS diet. *p < 0.05. (**C**,**D**) hAS^+/−^ mice had significantly higher gene expressions of the kidney sodium chloride co-transporter as compared to WT mice fed with either NS or HS diet. *p < 0.05. (**E**) HS diet increased hematocrit in hAS^+/−^ but not WT mice. *p < 0.05, between AS-TG groups; ^†^ *p* < 0.05, compared to AS-TG Chow group; ^§^ *p* < 0.05, compared to AS-TG HS group.

Furthermore, since both epithelial Na channel (ENaC) and Na^+^/Cl^−^ co-transporter (NCC) in kidney were responsible for Na reabsorption and regulated by aldosterone^22, 23^, we examined expression levels of these two genes in hAS^+/−^ and WT mice (Fig. 4). Again, as expected, in both NS and HS–fed mice, hAS^+/−^ mice showed significantly higher expression of three subunits of ENaC in kidney than WT mice. Additionally, HS significantly induced ENaC expression in both types of mice. Similarly, hAS^+/−^ mice showed significantly higher expression of NCC in kidney than WT mice and HS significantly induced NCC expression in both types of mice.

### Suppression of high-salt-induced hypertension in hAS^+/−^ mice by Fadrozole, an aldosterone synthase inhibitor

**Figure 5:**
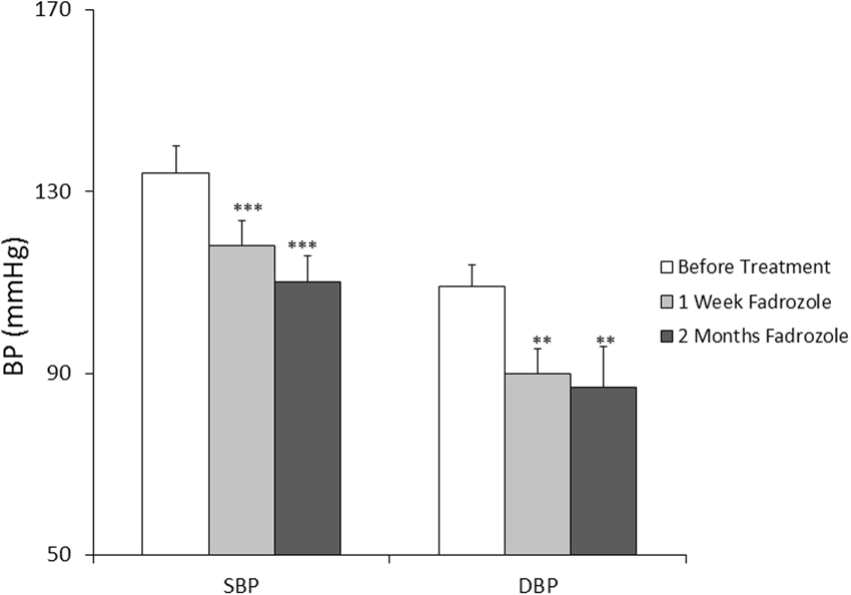
Fadrozole significantly suppressed high-salt-induced hypertension in hAS^+/−^ mice. hAS^+/−^ transgenic mice were fed with HS for 1 month and the BPs were measured. The mice on HS were treated continually with fadrozole for 2 months, the BPs of hAS^+/−^ mice were tested at the end of 1 week and 2 months treatment of fadrozole respectively. **p < 0.01; ***p < 0.001.

Aldosterone synthase (CYP11B2) inhibition has emerged as a new option for the treatment of hypertension. One week treatment of fadrozole (F3806, Sigma), an inhibitor of aldosterone synthase, at 4 mg/kg significantly reduced BPs of hAS^+/−^ mice with HS (SBP: 134 ± 6.1 mmHg versus 118 ± 5.5 mmHg, p < 0.001; DBP: 109 ± 4.8 mmHg versus 90 ± 5.3 mmHg, P < 0.01, n = 10/group) (Fig. 5). Continued treatment with fadrozole at this dose for 2 months further reduced BPs of hAS^+/−^ mice with HS (SBP: 110 ± 5.8 mmHg, p < 0.001; DBP: 87 ± 8.9 mmHg, P < 0.01: n = 10/group) (Fig. 5).

## Discussion

To determine the effects of hAS gene expression on BP, we established a transgenic mouse model that carries the human aldosterone synthase gene (cyp11B2) under the control of cyp11B1 promoter. Our study demonstrates that overexpression of hAS in mice results in significantly higher serum levels of aldosterone and high-salt-induced hypertension. We also observe that the high-salt-induced hypertension could be significantly reduced by the AS inhibitor, fadrozole, in these mice. These data suggest that AS overexpression plays the significant role in high dietary salt-induced BP in these mice.

Hypertension is one of the most important preventable causes of premature morbidity and mortality in the developed world. It is a key risk factor for ischemic and hemorrhagic stroke, myocardial infarction, heart failure, chronic kidney disease, and cognitive decline^24^. Aldosterone is a major mineralocorticoid hormone that plays a key role in the regulation of electrolyte balance and blood pressure. Excess aldosterone levels can arise from dysregulation of the renin-angiotensin-aldosterone system and are implicated in the pathogenesis of hypertension, chronic kidney disease and heart failure. It has been shown that aldosterone regulates Na^+^ conservation in the distal nephron through multiple mechanisms that modulate the activity of the epithelial Na^+^ channel (ENaC) including rapidly transcriptionally up-regulating the ENaCα subunit and indirectly regulating the ubiquitination of ENaC subunits^22^. Genetic deletion or loss-of-function mutations in the channels leads to Na wasting and hypotension, while conversely, hyperactivity of the channels causes Na retention and hypertension^25, 26^. Additionally, Na^+^ reabsorption is mediated by Na^+^/Cl^−^ co-transporter (NCC) that is also under the influence of aldosterone^23^. In this study, on either NS or HS diet, interestingly, expressions of three ENaC subunits and NCC in kidneys of the AS^+/−^ mice were significantly increased, accompanied by increased plasma aldosterone. The considerably increased BP of the AS^+/−^ mice on the HS diet probably results from a substantially increase in sodium reabsorption in the distal nephron consequent to the markedly increased expression of ENaC and NCC induced by elevated plasma aldosterone in these mice^27^.

In the AS^+/−^ mice, we observed markedly expressions of AS in brain. It is quite possible that HS-induced HP in the AS^+/−^ mice also results from an increased expressions of brain aldosterone. It has been shown that intracerebroventricular (icv) infusion of aldosterone does not change renal sympathetic nerve activity (RSNA), BP, or HR. However, modestly higher Na^+^ concentrations induce significant sympathetic and blood pressor responses^28^. It has been suggested that increased synthesis of aldosterone in the central nervous system contributes to salt sensitivity of BP by activation of its downstream receptor (mineralocorticoid receptor (MR))-epithelial Na^+^ channel (ENaC)-endogenous ouabain (EO) pathway^28^.

Aldosterone’s downstream MR blockers such as spironolactone and eplerenone block the actions of aldosterone at the receptor level. Clinical trials indicate that spironolactone and eplerenone effectively lower BP, particularly in resistant hypertension, and improving outcomes in congestive heart failure^29–34^. However, use of these agents, particularly spironolactone, can be limited by adverse effects, and is associated with reactive increases in circulating aldosterone levels that theoretically exacerbate the detrimental actions of aldosterone^35–37^. Therefore, inhibition of aldosterone synthesis in hypertension treatments represents a novel approach to decreasing exposure of the cardiovascular system to aldosterone, which may prove advantageous in terms of tolerability and/or cardiovascular and renal protection.

Aldosterone synthase (cytochrome P450 11B2, CYP11B2) is the sole enzyme responsible for the production of aldosterone in humans^38^ and recently was assumed to be a therapeutic target for aldosterone-related HP treatments. Recently, several approaches have been made to generate AS transgenic mice; however, these mice did not show the high-salt-induced hypertension phenotype. One transgenic mouse line overexpressed AS in the heart, showing coronary dysfunction but no difference of blood pressure between these mice and wild type mice^16^. Another mouse line (AS^hi/hi^) was generated by replacing the 3′ untranslated region of AS mRNA with that from a stable mRNA^17^. Plasma aldosterone did not differ significantly between WT and AS^hi/hi^ on the normal-salt diet but was significantly higher in AS^hi/hi^ than WT mice on the high-salt diet. The BP was modestly but significantly increased by10 mmHg in AS^hi/hi^ on the high-salt diet as compared to WT mice on the high-salt diet. It was noted that the BP was not significantly induced in AS^hi/hi^ on the high-salt diet as compared to AS^hi/hi^ on the normal-salt diet and the BP induced by the high-salt diet remained within the normal range (<120 mmHg,). In contrast, our hAS^+/−^ mice showed much higher serum level of aldosterone when they were fed with NS (~2 folds) and HS (~18 folds) as compared to WT mice fed with the same food. The BP in hAS^+/−^ mice on the HS diet was not only significantly increased as compared to WT mice on the HS diet, but also markedly induced as compared to the NS diet (135 vs. 100 in a one-month treatment and 137 vs. 104 in a three-month treatment). Nonetheless, this experimental AS mouse model appears to be more acceptable for future aldosterone-related hypertension research. Additionally, similar to Primary Hyperaldosteronism usually with lower hematocrit^39^, there were slightly but significant reduced hematocrit in NS fed hAS^+/−^ mice as compared to NS fed mice. Interestingly, consistent to the previous report^40^, HP induced by HS was accompanied with significantly increased hematocrit in hAS^+/−^ mice as compared to WT mice without induced HP.

We have for the first time demonstrated that hAS overexpression in Tg mice was involved in aldosterone overexpression and high salt-induced mouse HP. hAS was highly expressed in adrenal glands and hAS overexpression increased levels of aldosterone in hAS^+/−^ mice. Under the normal salt condition, the increase in hAS expression in the hAS^+/−^ mice had no significant effects on BP. In contrast, with high salt diet, BP of hAS^+/−^ mice was markedly increased as compared to wild type and normal salt-treated hAS^+/−^ mice. Additionally, we demonstrated that fadrozole treatments at 4 mg/kg, the effective dose that was used for blocking AS activity *in vivo* ^41^ significantly reduced the elevated BPs in the high salt-treated hAS^+/−^ mice. Our results suggest that this mouse model with hAS overexpression could be an appropriate animal model for AS and aldosterone overexpression/PA related hypertension study. Aldosterone synthesis may be an ideal pharmacological therapy target for aldosterone-related hypertension.

## Methods

### Vector Construction and Generation of Transgenic Mouse Lines

We set up a mouse colony by performing vasectomy, pronuclear injection of the CYP11B1-hAS fragment containing the human cyp11B1 promoter (pCYP11B1 (−2015–+3))^18^ and the human hAS coding sequence (cDNA, +4–+1515) (Fig. 1A) into a CH3 donor zygote, transfer of injected zygotes into recipient foster CH3 mice, as well as screening of offspring by Southern blot analysis of tail DNA to identify a mouse founder line. Transgenic offspring from each line were determined by PCR analysis of tail DNA using the following primers specific for hAS: 5′-GTG GCG TGT TCT TGT TGA ATG-3′ and 5′-GGT GGA TTT GAA CAT GAC CTC-3′ (generating a 300-bp product). All subsequent analyses were performed with heterozygous transgenic mice.

### Mice

Mice were bred in the laboratory of Animal Center at Indiana University School of Medicine. Mice were housed 3–5 per cage, fed with food and water ad libitum, and maintained in a 12-h light/dark cycle facility. Animal protocols pertinent to this study were approved by the Indiana University School of Medicine Laboratory Animal Resource Center.

### RNA isolation, reverse transcription-PCR

Total RNA was extracted using RNeasy mini kit (Qiagen, Valencia, CA, USA) and reverse transcribed by M-MLV (Invitrogen, Carlsbad, CA, USA). Derived complementary DNAs were amplified using PCR master mix (Promega, Madison, WI, USA). Primer sequences for hAS were as follow: 5′-GTG GCG TGT TCT TGT TGA ATG-3′ and 5′-GGT GGA TTT GAA CAT GAC CTC-3′. β-actin was amplified as a reference gene with primers 5′-ACCGCTCGTTGCCATTAGTGATGA-3′ and 5′-AAGGCCAACCGTGAAAAGATGACC-3′.

### Quantification of Aldosterone and Corticosterone in serum and urine by ELISA assay

Aldosterone levels in serum and urine and corticosterone levels in serum were measured using enzyme-linked immunosorbent assay (ELISA) kits (Aldosterone: ab136933, Abcam, Cambridge, MA; Corticosterone: 55-CORMS-E01, Alpco, Salem, NH), according to the manufacturer’s instructions.

### BP measurements

Mean systolic (SBP) and diastolic blood pressures (DBP) of conscious mice were measured using a CODA tail-cuff non-invasive blood pressure system (Model CODA6, Kent Scientific, Torrington, CT) following the manufacturer’s specifications. Before measurement, animals were conditioned by exposed to the environment and instrument on a daily basis for a week. A warmed restraining chamber was used to maintain mice, an inflatable occlusion cuff was placed around the mouse tail, as well as a volume pressure recording cuff was used according to the manufacturer’s specifications to measure arterial systolic pressure, arterial diastolic pressure, and heart rate. The blood pressure of trained mice was monitored starting at 2 pm for 30–40 min and the mean value of 5 final readings were obtained after 15 initial consecutive readings whose values were within 5% of the mean. Basal blood pressure was determined in NS mice fed with a normal diet containing 0.2% NaCl (Harlan Teklad, 2018) and HS mice that were switched to a high salt diet (high-salt chow containing 4% NaCl, Harlan Teklad, 2018) allowed to equilibrate at least for 1 week on the diet. Mice were allowed free access to tap water at all times.

### Statistical Analysis

One-way analysis of variance (ANOVA) was used for statistical analyses and comparisons for the differences between groups. All data are expressed as mean ± standard error of the mean (SEM). Differences between two means were considered significant when p was at least equal or less than 0.05.
