# Author Correction: Salt-dependent Blood Pressure in Human Aldosterone Synthase-Transgenic Mice

**DOI:** 10.1038/s41598-018-31443-0

**Published:** 2018-10-25

**Authors:** Huiying Gu, Zhizhong Ma, Jian Wang, Timothy Zhu, Nicole Du, Adam Shatara, Xin Yi, Mark C. Kowala, Yansheng Du

**Affiliations:** 1 Department of Neurology, Indiana University School of Medicine, Indianapolis, IN USA; 2 Basic Medical School, Peking University, Beijing, China; 3 Lilly Research Laboratories, Eli Lilly and Company, Indianapolis, IN USA

Correction to: *Scientific Reports* 10.1038/s41598-017-00461-9, published online 28 March 2017

The Acknowledgments section of this Article is incomplete, where:

“Jian Wang is deceased. This paper is dedicated to his memory”

should read:

“This research was supported by the Lilly research fund and the National Natural Science Foundation of China (Grant No. 81541009). Jian Wang is deceased. This paper is dedicated to his memory”

